# Estimation of Sex From the Buccolingual Dimension of Tooth Among the North Indian Population

**DOI:** 10.7759/cureus.58495

**Published:** 2024-04-17

**Authors:** Mohammad Abdurrahman Khan, Manisha Verma, Pratibha Dwivedi, Syed Belal Hassan, Anoop K Verma

**Affiliations:** 1 Department of Forensic Medicine and Toxicology, Hind Institute of Medical Sciences, Lucknow, IND; 2 Department of Periodontology, King George's Medical University, Faculty of Dental Sciences, Lucknow, IND; 3 Department of Anatomy, Hind Institute of Medical Sciences, Lucknow, IND; 4 Department of Community Medicine, Integral Institute of Medical Sciences & Research, Integral University, Lucknow, IND; 5 Department of Forensic Medicine and Toxicology, King George's Medical University, Lucknow, IND

**Keywords:** anthropometric, measurement, buccolingual, odontometric, dimension, tooth, sexual dimorphism, sex estimation

## Abstract

Introduction: Identification is an important aspect of forensic medicine. Identification plays an imperative role, especially in highly decomposed bodies, mutilated bodies, and undisclosed and fragmentary human remains. The estimation of sex is an essential parameter of human identification. In forensic anthropometry, sex determination is related to morphometric characteristics of skeletal bones, such as the skull and mandible, clavicle, sternum, scapula, humerus, pelvic bone, sternum, and femur. Since teeth are decay-resistant, conscientious analysis of teeth can accredit reliable sex estimation of an individual, especially when other determinants are fragmented or destroyed.

Aim: The aim of the study was to explore the association between sex and buccolingual crown dimensions of teeth.

Materials and methods: The study sample consists of 100 volunteer subjects (50 male subjects and 50 female subjects) aged between 20 and 35 years. Alginate was used to take impressions of the teeth and the cast was prepared using pouring by dental stone. Measurements of buccolingual parameters of all the teeth (except the third molars) of both jaws were done on dental casts by using a digital caliper.

Results: Collected data were analyzed using SPSS software (IBM Corp., Armonk, NY) and were outlined as mean and standard deviation (SD). The male and female groups were compared using an independent Student’s t-test or unpaired test. The results of this study revealed that 16 out of 28 odontometric parameters (except third molar) of the two groups (male and female) were higher in the male group as compared to the female group (p < 0.05).

Conclusion: Buccolingual odontometric parameters can be used for sex estimation in the North Indian population.

## Introduction

Identification is an important aspect of forensic medicine. Identification plays an imperative role, especially in decomposed bodies, skeletal remains, severely injured bodies, and dismembered bodies [1]. The estimation of sex is an essential parameter of human identification [2,3]. To create a biological profile, various methods of identification are used for an unknown person. Among various methods, sex estimation from the body remains is an important part of the forensic identification of unknown persons [1]. Sex prediction makes identification easy because a missing person of estimated sex is considered [4]. Sex determination also gives knowledge about the evolution, life history, and behavior of a particular population [5]. In forensic anthropometry, sex determination is related to morphometric characteristics of the different bones in the human body [6]. Among the skeleton, the pelvic bone is the most reliable bone for sex estimation with an accuracy of more than 95%. Craniofacial and mandibular bones are also reliable for sex determination with high accuracy [5]. Analysis of DNA gives definitive proof of sex determination, but such methods are exhaustive and relatively prolonged [7].

A difficulty may arise when there is an advanced decomposed body, a deliberately mutilated body with fragmentary remains, a broken jaw with some teeth, a skull with few teeth, or a body destroyed in a massive burn, mass disaster, railway accident, massive road traffic accident, or airplane crash [1]. Bones tend to break down after deposition, thus creating difficulties in their assessment, particularly in mass disasters or catastrophic events, where these bones rarely appear complete [5].

Since teeth are decay-resistant, conscientious analysis of teeth can accredit reliable sex estimation of an individual, especially when other determinants are fragmented or destroyed [8]. Hence, teeth can be scrutinized as a utilitarian research tool for such scenarios, since there is a significant chance to recover them intact from the remains of the bony skeleton, hence they play a dominant role when other variables of the bony skeleton are unavailable [5]. A tooth can be used to determine the sex of an individual on the basis of morphology, dimension, dissimilarity in dental development and eruption pattern, and expression of enamel protein amelogenin [5,8]. The aim of our study was to look for statistically significant gender differences in buccolingual crown dimensions.

## Materials and methods

Material required

The material required for the study included a spatula, rubber bowl, dental stone, alginate, mandibular, and maxillary impression tray.

**Figure 1:**
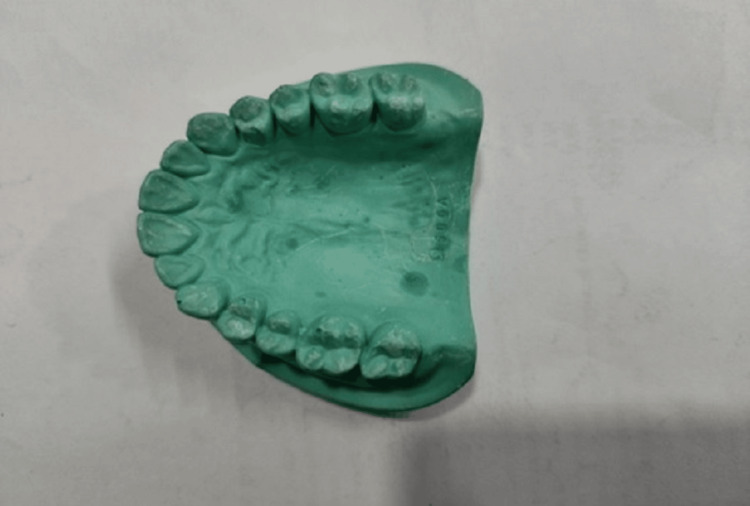
Image of a dental cast

Alginate is an irreversible, elastic hydrocolloid material, which forms an impression (negative mold) of teeth, which can be used for making a cast (positive mold) of the dentition of a patient (Figure 1).

Sample size determination

The sample size for the study was derived from the following formula: n = Zα2 p(1-p)/E2. Where E is the permissible error, p is proportion, Zα is the value of standard normal distribution at α level of significance (at α= 5%, Zα = 1.96), and n is the sample size. An accuracy of 63% was observed by the maxillary buccolingual dimension while an accuracy of 68.9% was observed by the mandibular buccolingual dimension, so the average accuracy for the buccolingual dimension was 66.4% [9]. So, p = 66.4%, i.e., .664, 1-p = .336, and E = 9%, i.e., .09. So, n = (1.96) 2 × 0.664 × 0.336/(.09)2 = 105.8 ≈ 100.

The present study was a cross-sectional study conducted at Integral Institute of Medical Sciences & Research (IIMS&R), Integral University, Lucknow. The study sample consists of 100 volunteer subjects (50 male subjects and 50 female subjects) aged between 20 and 35 years. Informed consent (written) was taken from all subjects before taking dental impressions. Institutional ethical clearance was taken before starting the study with reference number IEC/IIMSR/2023/12.

Inclusion criteria

The inclusion criteria are shown in Table 1.

**Table 1:** Inclusion criteria

S. No.	Inclusion
1.	Subjects aged between 20 and 35 years
2.	No previous orthodontics treatment or any history of prosthesis
3.	Complete and fully erupted teeth
4.	Periodontally healthy teeth

Exclusion criteria

There are various exclusion criteria, which are shown in Table 2.

**Table 2:** Exclusion criteria

S. No.	Exclusion
1.	Spacing teeth, misaligned teeth, diastema, or crowded teeth
2.	Restored teeth, mobile teeth
3.	Third molar (third molar was excluded from the study because most of the time it is impacted or unerupted and causes issues with oral health)
4.	Carious teeth
5.	Attrited teeth
6.	Traumatic teeth or fractured teeth
7.	Hypoplastic teeth

Methodology and tooth measurements

**Figure 2:**
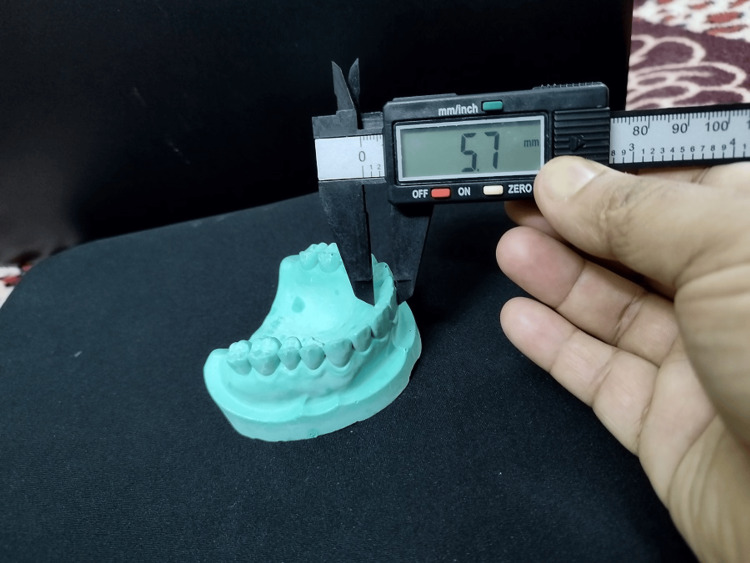
Measurement of the buccolingual parameter of teeth using a digital caliper

Alginate was used to take an impression of the teeth and the cast was prepared using pouring by dental stone (Figure 1). Measurements of buccolingual (BL) parameters of all the teeth (except the third molars) of both jaws were done on dental casts by using a digital caliper, which was calibrated to 0.01 mm. Buccolingual width is the distance between the buccal or labial surface and the palatal or lingual surface of the crown of the teeth, which is measured using the caliper held at right angles to the mesiodistal dimension of teeth (mesiodistal dimension is the maximum distance between approximate contact point on crown surface of the teeth) (Figure 2).

Measurement of misaligned or rotated teeth was done by placing the caliper between the approximate contact point on the crown surface of the teeth.

## Results

Collected data were analyzed using SPSS software (IBM Corp., Armonk, NY) and were outlined as mean and standard deviation (SD). The male and female groups were compared using two independent sample t-tests. The results of this study revealed that five out of seven odontometric buccolingual parameters of the upper right jaw (Table 3) of the two groups (male and female) showed statistically significant differences (p-value < 0.05), three out of seven odontometric buccolingual parameters of the upper left jaw (Table 4) of the two groups (male and female) showed statistically significant difference (p-value < 0.05), four out of seven odontometric buccolingual parameters of the lower right jaw (Table 5) of the two groups (male and female) showed statistically significant difference (p-value < 0.05), and four out of seven odontometric buccolingual parameters of the lower left jaw (Table 6) of the two groups (male and female) showed statistically significant difference (p-value < 0.05). However, for the mean of the rest of the parameters, two out of seven odontometric buccolingual parameters of the upper right jaw (Table 3) of the two groups (male and female) did not show a significant difference among both sex groups (p-value > 0.05), four out of seven odontometric buccolingual parameters of the upper left jaw (Table 4) of the two groups (male and female) did not show significant difference among both sex groups (p-value > 0.05), three out of seven odontometric buccolingual parameters of the lower right jaw (Table 5) of the two groups (male and female) did not show significant difference among both sex groups (p-value > 0.05), and three out of seven odontometric buccolingual parameters of the lower left jaw (Table 6) of the two groups (male and female) did not show significant difference among both sex groups (p-value > 0.05).

**Table 3:** Comparing mean and standard deviation between males and females for upper right jaw buccolingual parameter

Upper right jaw buccolingual parameters	Male		Female		P-value
	Mean (mm)	SD	Mean (mm)	SD	
Upper right jaw buccolingual central incisor	7.22	0.53	6.98	0.48	0.021
Upper right jaw buccolingual lateral incisor	6.49	0.64	6.59	1.07	0.55
Upper right jaw buccolingual canine	8.07	0.78	7.71	0.45	0.006
Upper right jaw buccolingual 1st premolar	9.18	0.48	8.76	0.66	<0.001
Upper right jaw buccolingual 2nd premolar	9.23	0.64	8.93	0.66	0.026
Upper right jaw buccolingual 1st molar	11.03	0.64	10.67	0.46	0.002
Upper right jaw buccolingual 2nd molar	10.92	0.81	10.73	0.45	0.165

**Table 4:** Comparing mean and standard deviation between males and females for upper left jaw buccolingual parameter.

Upper left jaw buccolingual parameters	Male		Female		P-value
	Mean (mm)	SD	Mean (mm)	SD	
Upper left jaw buccolingual central incisor	7.22	0.59	6.87	0.46	0.001
Upper left jaw buccolingual lateral incisor	6.49	0.56	6.30	0.42	0.06
Upper left jaw buccolingual canine	8.02	0.83	7.73	0.55	0.045
Upper left jaw buccolingual 1st premolar	9.14	0.62	8.98	0.60	0.199
Upper left jaw buccolingual 2nd premolar	9.20	0.59	9.01	0.51	0.082
Upper left jaw buccolingual 1st molar	10.98	0.56	10.71	0.42	0.008
Upper left jaw buccolingual 2nd molar	12.05	0.43	12.65	0.40	0.796

**Table 5:** Comparing mean and standard deviation between males and females for lower right jaw buccolingual parameter

Lower right jaw buccolingual parameters	Male		Female		P-value
	Mean (mm)	SD	Mean (mm)	SD	
Lower right jaw buccolingual central incisor	5.90	0.55	5.56	0.36	<0.001
Lower right jaw buccolingual lateral incisor	5.96	0.45	5.83	0.35	0.108
Lower right jaw buccolingual canine	7.46	0.70	6.86	0.90	<0.001
Lower right jaw buccolingual 1st premolar	7.81	0.66	7.54	0.49	0.021
Lower right jaw buccolingual 2nd premolar	8.35	0.52	8.19	0.45	0.119
Lower right jaw buccolingual 1st molar	10.04	1.85	10.16	0.67	0.672
Lower right jaw buccolingual 2nd molar	10.20	0.47	9.88	0.41	0.001

**Table 6:** Comparing mean and standard deviation between males and females for lower left jaw buccolingual parameter

Lower left jaw buccolingual parameters	Male		Female		P-value
	Mean (mm)	SD	Mean (mm)	SD	
Lower left jaw buccolingual central incisor	5.77	0.50	5.57	0.39	0.026
Lower left jaw buccolingual lateral incisor	5.96	0.39	5.88	0.38	0.296
Lower left jaw buccolingual canine	7.43	0.71	6.98	0.69	0.002
Lower left jaw buccolingual 1st premolar	7.88	0.61	7.58	0.53	0.011
Lower left jaw buccolingual 2nd premolar	8.33	0.58	8.30	0.73	0.819
Lower left jaw buccolingual 1st molar	10.48	0.53	10.26	0.41	0.022
Lower left jaw buccolingual 2nd molar	10.18	0.57	9.85	1.27	0.101

The effect size for a t-test for an independent sample was calculated using Cohen's d. Effect size = Xm-Xf/SD, where Xm and Xf are the mean values for males and females, respectively, and SD is the mean standard deviation. Using this formula, we calculated the effect size. For the upper right jaw, the buccolingual parameter was 0.4047, for the upper left jaw, the buccolingual parameter was 0.2488, for the lower right jaw, the buccolingual parameter was 0.3853, and for the lower left jaw, the buccolingual parameter was 0.3884.

Sexual dimorphism was calculated using the following formula [10]: % sexual dimorphism = [(Xm/Xf)-1] × 100, where Xm and Xf are the mean values for males and females, respectively. Sexual dimorphism in both upper and lower jaws for all 16 significant odontometric parameters is shown in Tables 7-10.

**Table 7:** Sexual dimorphism using upper right jaw buccolingual parameters

S. No.	Upper right jaw buccolingual parameters	% Sexual dimorphism
1.	Upper right jaw buccolingual central incisor	3.43
2.	Upper right jaw buccolingual central incisor	4.66
3.	Upper right jaw buccolingual central incisor	4.79
4.	Upper right jaw buccolingual central incisor	3.35
5.	Upper right jaw buccolingual central incisor	3.37

**Table 8:** Sexual dimorphism using upper left jaw buccolingual parameters

S. No.	Upper left jaw buccolingual parameters	% Sexual dimorphism
1.	Upper left jaw buccolingual central incisor	5.09
2.	Upper left jaw buccolingual canine	3.75
3.	Upper left jaw buccolingual 1st molar	2.52

**Table 9:** Sexual dimorphism using lower right jaw buccolingual parameters

S. No.	Lower right jaw buccolingual parameters	% Sexual dimorphism
1.	Lower right jaw buccolingual central incisor	6.11
2.	Lower right jaw buccolingual canine	8.74
3.	Lower right jaw buccolingual 1st premolar	3.58
4.	Lower right jaw buccolingual 2nd molar	3.23

**Table 10:** Sexual dimorphism using lower left jaw buccolingual parameters

S. No.	Lower left jaw buccolingual parameters	% Sexual dimorphism
1.	Lower left jaw buccolingual central incisor	3.59
2.	Lower left jaw buccolingual canine	6.44
3.	Lower left jaw buccolingual 1st premolar	3.95
4.	Lower left jaw buccolingual 1st molar	2.14

## Discussion

Since teeth are decay-resistant and most durable, much of the research has been done on teeth in the past [11-13]. Various studies have been done on dental morphology, dental pathology, and odontometric variation but forensic expert focused their research on sex estimation and age estimation [8]. Dental sexual dimorphism has been addressed for long and many research studies have shown that odontometric parameters can be used perfectly to estimate the sex in living and skeleton remains [14-16]. In this study, the buccolingual (BL) dimension of all teeth (except third molars) was used to determine the sex of an individual. On measurement (Table 3) among upper right jaw parameters, buccolingual central incisor, buccolingual canine, buccolingual second premolar, and buccolingual first molar were statistically significant while buccolingual first premolar of the right upper jaw parameter was statistically highly significant and different in male compared to female groups. Among upper left jaw parameters (Table 4), buccolingual central incisor, buccolingual canine, and buccolingual first molar were statistically significant in the male group compared to the female group. Among lower right jaw parameters (Table 5), the buccolingual dimensions of the first premolar and second molar were statistically significant and different in the male group compared to the female groups while buccolingual central incisor and buccolingual canine were highly significant and different in the male group compared to the female group. Among lower left jaw parameters (Table 6), buccolingual central incisor, buccolingual canine, buccolingual first premolar, and buccolingual first molar were statistically significant and different in male groups compared to female groups. In a study done by Ahn et al. [17], in the upper jaw first premolar, second premolar, and second molar, lower jaw canine, first premolar, and second molar, the overall accuracy for sexual dimorphism was relatively higher in the odontometric parameters used to differentiate males and females. Lower jaw odontometric parameters of lateral incisor and second molar were given low priority for sex determination as compared to other teeth measurements as they had somewhat low accuracy for all odontometric measurements [17]. Our study showed that in comparison between the two sexes, the accuracy of all parameters was higher for males. This result is similar to studies done by Sharma et al. [9] and Kazzazi et al. [18]. Our study demonstrates that among buccolingual dimensions of upper jaw teeth, the upper right jaw teeth dimension was better than upper left jaw teeth dimensions for sex estimation. Among the buccolingual dimensions of lower jaw teeth, the lower right jaw teeth and lower left jaw teeth dimensions had equal values for sex estimation. Our study also reveals that among upper jaw and lower jaw, lower jaw odontometric parameters were better for sex estimation, which was similar to Angadi et al. who measured both mesiodistal and buccolingual dimensions of all teeth except third molars in the Indian population and found that canine was most dimorphic teeth (lower canine more than upper canine) [19]. Our study reveals maximum sexual dimorphism shown by lower right jaw (mandibular) buccolingual canine (8.74%), followed by lower left jaw (mandibular) buccolingual canine (6.44%), which was similar to the finding of Angadi et al. [19]. The present study shows that when canine width is higher than 7.188 mm, the possibility of an individual being male is 100% whereas a study done by Kaushal et al. [20] on the Indian population shows that when the canine width is higher than 7 mm, the chance of the subject being a male is 100%. Another study done on a group of Lebanese adults indicates the probability of estimation of sex with an accuracy of 95%, showing when the mean canine width was greater than 7.188 mm, the sex was male [21].

There are certain limitations of the study. As the sample would not be representative of the entire population, there may be a chance of bias during analysis. Being a small study population, the accuracy percentage obtained may be a little bit inflated. Moreover, the odontometric parameters may be different in different geographical conditions and may affect the analysis. Sex estimation will be difficult if teeth recovered from decomposed bodies or mutilated bodies have certain pathologies or fractures.

## Conclusions

Estimation of sex is one of the important parameters in forensics for establishing the identity of an individual in mutilated, highly decomposed, undisclosed, fragmentary human remains or skeleton remains. The present study revealed that in 16 out of 28 odontometric parameters of the two groups (male and female), the t-test showed statistically significant and different results in the male group compared to the female group. Maximum sexual dimorphism is shown by the lower right jaw (mandibular) buccolingual canine (8.74%), followed by the lower left jaw (mandibular) buccolingual canine (6.44%). So, sex can be determined by buccolingual teeth measurements. Since these parameters are population-specific, they may not be applicable to all populations.
